# Supplementary material for: Pharmacy Staff Experiences and Needs During Second Dispense of Driving-Impairing Medicines: A Qualitative Study
Source: Pharmacy (Basel). 2025 Oct 9;13(5):146. doi: 10.3390/pharmacy13050146 (PMC12567210; doi:10.3390/pharmacy13050146)
Supplement: Supplementary file 1 [file pharmacy-13-00146-s001.zip › pharmacy-3771731-supplementary.pdf]

Supplementary File S1: COREQ checklist

K. Benning, L. van Dijk, J.J. de Gier and S.D. Borgsteede. Pharmacy Staff Experiences and Needs During Second Dispense of Driving-Impairing Medicines: A Qualitative Study. Pharmacy 2025.

Consolidated criteria for reporting qualitative studies (COREQ): 32-item checklist

Developed from:
Tong A, Sainsbury P, Craig J. Consolidated criteria for reporting qualitative research (COREQ): a 32-item checklist for interviews and focus groups. International Journal for Quality in Health Care. 2007. Volume 19, Number 6: pp. 349 – 357

Item No	Guide Questions/Description	Reported on Page #
Domain 1: Research team and reflexivity		
Personal Characteristics		
1. Interviewer/ facilitator	Which author/s conducted the interview or focus group?	4 (2.3)
2. Credentials	What were the researcher's credentials? E.g., PhD, MD	6 (2.5)
3. Occupation	What was their occupation at the time of the study?	6 (2.5)
4. Gender	Was the researcher male or female?	6 (2.5)
5. Experience and training	What experience or training did the researcher have?	4 (2.3)
Relationship with participants		
6. Relationship established	Was a relationship established prior to study commencement?	4 (2.2)
7. Participant knowledge of the interviewer	What did the participants know about the researcher? e.g. personal goals, reasons for doing the research?	4 (2.3)
8. Interviewer characteristics	What characteristics were reported about the interviewer/facilitator? e.g. Bias, assumptions, reasons and interests in the research topic	6 (2.5)
Domain 2: study design		
Theoretical framework		

Item No	Guide Questions/Description	Reported on Page #
9. Methodological orientation and Theory	What methodological orientation was stated to underpin the study? e.g. grounded theory, discourse analysis, ethnography, phenomenology, content analysis	6 (2.5), 11 (8)
Participant selection		
10. Sampling	How were participants selected? e.g., purposive, convenience, consecutive, snowball	4 (2.2)
11. Method of approach	How were participants approached? e.g., face-to-face, telephone, mail, email	4 (2.2)
12. Sample size	How many participants were in the study?	4 (2.2), 6 (3)
13. Non-participation Setting	How many people refused to participate or dropped out? Reasons?	4 (2.2)
14. Setting of data collection	Where was the data collected? e.g., home, clinic, workplace	4 (2.3)
15. Presence of nonparticipants	Was anyone else present besides the participants and researchers?	4 (2.3)
16. Description of sample	What are the important characteristics of the sample? e.g. demographic data, date	6-7 (3)
Data collection		
17. Interview guide	Were questions, prompts, and guides provided by the authors? Was it pilot tested?	5 (2.4)
18. Repeat interviews	Were repeat interviews carried out? If yes, how many?	n.a.
19. Audio/visual recording	Did the research use audio or visual recording to collect the data?	4 (2.3)
20. Field notes	Were field notes made during and/or after the interview or focus group?	4 (2.3)
21. Duration	What was the duration of the interviews or focus group?	6 (3)
22. Data saturation	Was data saturation discussed?	14 (7.1)
23. Transcripts returned	Were transcripts returned to participants for comment and/or correction?	14 (7.1)
Domain 3: analysis and findings		
Data analysis		

Item No	Guide Questions/Description	Reported on Page #
24. Number of data coders	How many data coders coded the data?	5 (2.5)
25. Description of the coding tree	Did the authors provide a description of the coding tree?	5-6 (2.5), 7 (3), Supplementary file 2
26. Derivation of themes	Were themes identified in advance or derived from the data?	5-6 (2.5), Supplementary file 2
27. Software	What software, if applicable, was used to manage the data?	6 (2.5)
28. Participant checking	Did participants provide feedback on the findings?	5 (2.5)
Reporting		
29. Quotations presented	Were participant quotations presented to illustrate the themes/findings? Was each quotation identified? e.g., participant number	7-11 (4.1-6.2)
30. Data and findings consistent	Was there consistency between the data presented and the findings?	7-11 (4.1-6.2)
31. Clarity of major themes	Were major themes clearly presented in the findings?	6-11 (4-6.2), Figure 1.
32. Clarity of minor themes	Is there a description of diverse cases or a discussion of minor themes?	11-15 (7)

Supplementary File S2: coding tree

Main Theme	Description
First dispense consultation - information (D[1])	Provided verbal and written information provided by pharmacy staff
First dispense consultation – deliveries (D)	How do patients whose medicines are delivered or placed in the pick-up locker receive the information
Second dispense consultation - information (D)	Provided verbal and written information provided by pharmacy staff
Second dispense consultation - deliveries (D)	How do patients whose medicines are delivered or placed in the pick-up locker receive the information
Second dispense consultation – experiences/side effects (D)	What are the patients’ experiences when using the medicine
Differences between medication on fitness to drive (D)	Is a distinction made between category I, II, and III in terms of explanation
Use of supporting tools (D)	Which supporting tools are used by pharmacy staff
Structure in conversations (I[2])	How structure is applied in second dispense conversations
Dialogue (I)	How assistants ensure they have a dialogue with patients
Barriers during second dispense (D)	Which factors create barriers during second dispense
Trust (I)	Enough trust to address private matters during second dispense
Most important for a second dispense conversation (D)	What is most important for a good second dispense
Solutions for barriers (D)	What are solutions for the barriers
Vehicles mentioned during dispense (D)	Which vehicles are mentioned during dispensing of driving-impairing medicines
Difference between first and second dispense (D)	Difference between second dispense of driving-impairing medicines and a medicine that does not affect driving ability
Bringing up driving ability themselves (I)	Whether patients bring up driving ability themselves during second dispense
What patients appreciate (D)	What patients appreciate you asking about, or what they do not appreciate
Second dispense guidance at the moment (D)	Pharmacy staff view on second dispense guidance for driving-impairing medicines
Needs during second dispense (D)	What needs during second dispense for driving-impairing medicines
Ideal second dispense (D)	What an ideal second dispense for driving-impairing medicines looks like
TRIAGE questions	What pharmacy staff think of the example TRIAGE questions.

[1] D: result of Deductive coding.
[2] I: result of Inductive coding.
